# The Effect of COVID-19 Restrictions on Routine Activities and Online Crime

**DOI:** 10.1007/s10940-022-09564-7

**Published:** 2022-12-08

**Authors:** Shane D. Johnson, Manja Nikolovska

**Affiliations:** Dawes Centre for Future Crime at University College London, London, WC1H 9EZ UK

**Keywords:** Online fraud, Online hacking, Doorstep fraud, Routine activity theory, COVID-19

## Abstract

**Objectives:**

Routine activity theory suggests that levels of crime are affected by peoples’ activity patterns. Here, we examine if, through their impact on people’s on- and off-line activities, COVID-19 restriction affected fraud committed on- and off-line during the pandemic. Our expectation was that levels of online offending would closely follow changes to mobility and online activity—with crime increasing as restrictions were imposed (and online activity increased) and declining as they were relaxed. For doorstep fraud, which has a different opportunity structure, our expectation was that the reverse would be true.

**Method:**

COVID-19 restrictions systematically disrupted people’s activity patterns, creating quasi-experimental conditions well-suited to testing the effects of “interventions” on crime. We exploit those conditions using ARIMA time series models and UK data for online shopping fraud, hacking, doorstep fraud, online sales, and mobility to test hypotheses. Doorstep fraud is modelled as a non-equivalent dependent variable, allowing us to test whether findings were selective and in line with theoretical expectations.

**Results:**

After controlling for other factors, levels of crime committed online were positively associated with monthly variation in online activities and negatively associated with monthly variation in mobility. In contrast, and as expected, monthly variation in doorstep fraud was positively associated with changes in mobility.

**Conclusions:**

We find evidence consistent with routine activity theory, suggesting that disruptions to people’s daily activity patterns affect levels of crime committed both on- and off-line. The theoretical implications of the findings, and the need to develop a better evidence base about what works to reduce online crime, are discussed.

**Supplementary Information:**

The online version contains supplementary material available at 10.1007/s10940-022-09564-7.

## Introduction

While urban crime has generally been decreasing in recent decades (Farrell and Birks 2018; Van Dijk et al. 2012; Zimring 2006), crime committed online has been on the rise (Caneppele and Aebi 2019; Levi et al. 2016). Various theories exist to explain why crime occurs, but relative to crime committed in physical environments, their application to crime committed online has received relatively less attention (for exceptions, see Leukfeldt and Yar 2016; Miró-Llinares and Moneva 2019; Yar 2005), particularly in terms of empirical testing. To simplify perspectives, some focus on explanations at the level of the offender and their peers, placing little importance on the role of crime opportunity, while others focus on the latter, taking the existence of motivated offenders as given. In 2020, governments implemented policies to address the COVID-19 pandemic that created conditions—otherwise rarely experienced by most people—that had the potential to influence crime opportunity on an unprecedented scale. This presented an unusual opportunity to test theories of crime (opportunity), as well as understand how large-scale disruptions such as pandemics affect crime.

Here, we do so with a focus on crime committed online. More specifically, we analyze patterns for three different types of fraudulent offenses (in the UK), selected because they are committed in high volumes and because they represent different types of offending: (1) online shopping and auction frauds (hereafter, online shopping fraud) which involve “sellers” falsely representing goods or not supplying them when purchased, or “buyers” using fraudulent means to purchase them (e.g. using stolen credit cards); (2) the hacking of people’s social media, email or other personal online accounts, which involve the unauthorized modification of a person’s social media or other accounts; and, (3) door-to-door sales and bogus tradesmen offenses (hereafter, doorstep crime), which involve a fraudster visiting a victim’s home to defraud them out of money (e.g. charging for services or goods not provided). This third offence type is important in its own right, but is included here because, as we will discuss below, the crime opportunity (but not motivation) for this type of offense is different to the online offenses analyzed. The paper is organized as follows. In the next section, we discuss the theoretical framing of the paper and studies that have examined the impact of the pandemic on urban crime. This is followed by a review of studies that have examined the impact of the pandemic on crime committed online, and then a description of our study design and hypotheses. We subsequently present our methodology and report our results. The paper concludes with a discussion of our findings and their implications.

### Background

Routine activity theory (Cohen and Felson 1979) focuses on the role of people’s activities and how these create crime opportunities by influencing the likelihood that a motivated offender will encounter a suitable target absent a capable guardian. Few would have predicted the dramatic shift to online working and “living” observed as a consequence of COVID-19 pandemic containment policies imposed through government restrictions (hereafter COVID-19 restrictions). Routine activities were disrupted at the individual and collective levels. For example, in most countries, mobility patterns decreased dramatically (Nivette et al. 2021), and many organizations pivoted from requiring staff to work in offices to allowing them to work from home (ILO 2020). Not surprisingly then, at the beginning of the pandemic many scholars (Ashby 2020a, b; Stickle and Felson 2020, Halford et al. 2020; Gerell et al. 2020) noted that these changes to routine activity patterns could directly affect crime opportunity, either by reducing the likelihood that offenders would encounter suitable targets in urban settings, or by increasing the amount of time people would spend at home and hence provide guardianship there. One of the first empirical studies to investigate this was conducted by Ashby (Ashby 2020a, b), who examined changes to urban crimes including assault, robbery, burglary and vehicle crime. Using time series data for 16 cities/counties in the US for the period Jan 2016 to May 2020, he found mixed results with reductions observed for some crime types in some cities but increases were noted in others. Given that the study was conducted so early in the pandemic, when little data were available for analysis and containment policies were still in their infancy, the equivocal findings are perhaps not surprising. The study did, however, provide much needed intelligence about the association between changes that occurred as a result of COVID-19 and levels of urban crime at a time when this was important to policy makers and the police. Moreover, the study provided a template for analyzing such changes.

Approximately six months later, Langton et al. (2021) analyzed crime trends in England and Wales for the first half of the pandemic year. Their findings suggested that—with only a few exceptions (anti-social behavior and drug offenses)—most urban crime types (various forms of theft, burglary, robbery, shoplifting, criminal damage and arson, vehicle crime, violence and sexual offenses, public order and possession of weapons) exhibited a sharp decline during the first UK lockdown, with a gradual resurgence coincident with the relaxation of the measures. By the end of 2021, almost unequivocally, and in line with routine activity theory, researchers concluded that urban crime had declined during lockdown periods in different countries (Balmori de la Miyar et al. 2021; Calderon-Anyosa and Kaufman 2021; Campedelli et al. 2021; Estévez-Soto, 2021; Halford et al. 2020; Hodgkinson and Andresen 2020; Brantingham 2021; Mohler et al. 2020; Nivette et al. 2021; Payne et al. 2021; Rashid 2021; Yang et al. 2021).

In contrast to activities in the real world, online activity (including the use of online shopping platforms) increased during the pandemic, creating different expectations from a routine activity perspective about how crime committed online might change. The routine activity perspective (Cohen and Felson 1979) was originally developed to explain direct contact predatory crime, and crimes committed in urban environments and online clearly differ. However, they do share fundamental characteristics that underpin the routine activity perspective. That is, a crime event can only occur online (or offline) if an offender interacts with a suitable target in some way in the absence of capable guardianship. As noted by others (e.g. Miro-Llinares and Johnson 2008; Yar 2005), in online environments these interactions may involve direct contact through (say) online chat rooms or shopping platforms, or interactions may occur more indirectly when (for example) a victim opens malicious software (malware) sent to them via email or downloaded online. Just as in the real world, guardianship exists online but it is variable in both application and effectiveness. For example, forums and social media platforms have moderators who monitor content and interactions but they cannot monitor all activity, and the level of monitoring provided varies by platform. As another example, consider that to generate revenue, websites often host advertisements. Some of these may be fraudulent or the websites that they link to may host malware, and website owners vary in the extent to which they protect consumers by verifying the authenticity of the adverts that they host (e.g. Zarras et al. 2014; Bermudez-Villalva et al. 2020). Finally, guardianship may be offered by software, such as firewalls and antivirus packages (Leukfeldt and Yar 2016), but various packages exist, which vary in effectiveness, and these are ineffective against zero-day attacks (i.e. unknown vulnerabilities). Returning to our expectation in the current study, during the pandemic, we have no reason to believe that capable guardianship changed online, but as online activity did increase as a consequence of lockdown restrictions (see below), absent fundamental changes in people’s security habits, this may have increased the number of (suitable) targets online, elevating people’s exposure to risk and the opportunities for crime in online environments (Kemp et al. 2021). This would be expected to lead to an increase in crime committed online even if the number of motivated offenders remained constant over time. In the next section, we review existing research that has explored if and how online crime changed during the pandemic.

### Cybercrime During the Pandemic

To date, findings from most—but not all—analyses of crime committed online during the pandemic suggest that online offending increased. However, the data used and the methodological adequacy of these studies varies and so while reviewing them we highlight the issues with these studies as well as their conclusions. Before doing so, it is also important to note that while crimes committed online vary, most (but not all) of the literature has focused on online fraud or the hacking of online accounts.

In their study, Lallie et al. (2021) created a timeline of global COVID-19 cyber-attacks for the period mid-March to mid-May 2020 using data collected from open-source media. In summarizing these, the authors characterized cyber attacks as opportunistic offenses that targeted particular victims susceptible to them, often using social engineering and the COVID-19 crisis as a ‘hook’ to perform attacks. The attacks discussed included hacking, the distribution of malware, online financial fraud, phishing, and extortion. Their timeline suggested a loose correlation between government announcements and media stories about the pandemic and the timing of cyber-attacks. However, no statistical analysis was reported and the reliance on open-source data is unlikely to provide a complete picture of offending.

Mackey et al. (2020) analyzed social media (Twitter and Instagram) posts for the period February-May 2020 to identify content involving illicit COVID-19 product sales. They observed a first wave of questionable products for the period 3 March—4 April 2020 that consisted of immunity-boosting treatments, followed by a second wave of posts about suspicious testing kits for the period 6 March—10 April 2020. While their dataset contained over six million observations of COVID-19 related posts, only 1867 of these featured illicit goods, but the study highlighted the use of social media to advertize such products.

In a different study, Naidoo (2020) thematically analyzed ﻿185 unique online COVID-19 scam incident documents posted between Mid-March and Mid-April 2020 provided by *FraudWatch International (*a global online fraud and cybersecurity services company*)*. In line with opportunity theories of crime, Naidoo concluded that cybercriminals seemed to have adapted to exploit the situational factors that the pandemic presented such as peoples’ need for social connectivity, a switch to remote working, and increased engagement in other online activities including shopping. Moreover, they found that attackers were using tactics including emotional appeals associated with the pandemic (and the promise of financial assistance) as a hook to facilitate scams.

In their study, Georgiadou et al. (2022) used a survey methodology, but this time examined organisational security-readiness in remote working in countries across Europe in April 2020. In terms of attacks experienced during the period considered, one in five reported that they had experienced some kind of security threat, and the authors also reported that risk varied across different business sectors. However, their results were based on a relatively small non-representative survey sample of employees (n = 246), and the survey covered the pandemic period only. In fact, for all of the studies discussed so far, data were not analyzed for the period prior to the pandemic, and hence it is difficult to estimate the counterfactual and consequently whether there was an actual increase in cyberattacks, fake posts or victimization during the pandemic.

Hawdon et al. (2020) also used a victimisation survey to estimate rates of online victimisation but did so for the periods before and at the start of the pandemic. They reported no differences in victimization rates for the two periods and suggested that the changes observed in other studies (discussed below) may not reflect actual changes in risk but rather a change in reporting rates. However, it is important to note that Hawdon et al. also failed to find significant changes to people’s online routines (e.g. time spent shopping online) for the two periods. One reason for this is likely to be the fact that the “COVID-19 period” used was the 12-month period up to April 2020. Most researchers would not consider this to be a pandemic period particularly as significant changes to people’s routine activities did not emerge until March/April 2020. As such, it is perhaps unsurprising that the authors observed no differences for the two periods compared. The authors did, however, find that other routine activity variables (e.g. variation in exposure to risk) predicted cybervictimization for the two periods considered.

To date, only a few studies have used time-series data to examine changes in crime committed online. Among them is a study by Kemp et al. (2021) who examined monthly variation in crime reported to Action Fraud (the UK’s national reporting centre for fraud and cyber crime) for the first five months of the first UK lockdown. Using a univariate ARIMA time series model to forecast levels of crime during the pandemic, they found that, relative to the observed trend for the previous three years, offenses including fraud and hacking increased (in most but not all months) relative to expectation during the first three months of the lockdown. In contrast, door-step fraud, which has no online component did not. For some online offenses, levels then appeared to have declined. These findings are consistent with the routine activity perspective, however, the authors had only a small number of data points for the COVID-19 period, and there was substantial monthly variation in the crime counts. This makes it difficult to draw any firm conclusions regarding longer-term trends on the basis of this analysis. Moreover, the authors did not model changes to people’s activity patterns meaning that the study did not provide a direct test of routine activity theory.

In a more recent paper, Buil-Gil et al. (2021) used data concerning online crime committed in Northern Ireland for a longer time series (April 2015–April 2021). Using an Ordinary Least Squares (OLS) regression model, the authors examined whether crime increased online and whether changes were associated with the timing of lockdowns. They found that online crime increased during the lockdown but—after controlling for long-term trends in the data—found no association between the changes observed in crime and the timing of lockdowns. While the observed increase in offenses is consistent with routine activity theory, the absence of a correlation between the volume of crime and the timing of lockdowns is not. However, we note that their model specification provided only a crude test of routine activity (see below) and the use of an OLS model, for which the assumption of the independence of observations would be violated (time series data are not independent), may have been problematic.

Overall, the above studies generally suggest that crime committed online increased during the pandemic (although some could not establish this) and that patterns were generally (but not always) consistent with routine activity theory, but none establish causality. That is, none of the studies can rule out important threats to internal validity (Campbell 1963), such as the possibility that changes observed could be attributed to factors other than the pandemic and the impact this had on peoples’ routine activities. For example, that variables which captured the timing of each of the lockdowns in the Buil-Gil et al. study were not associated with variation in online crime reduces confidence in the argument that it was COVID-19 restrictions– through their impact on people’s routine activities—that led to increases in this type of offending during the pandemic. Moreover, as these studies do not examine changes to routine activities directly, they leave unanswered the question as to what was the actual mechanism through which the lockdowns might have brought about their effects on crime committed online? Here, we test the routine activity explanation—that COVID-19 restrictions led to changes in peoples’ activity online or their mobility, which subsequently affected their risk of victimization—using a more nuanced approach than has been used in studies to date.

### The Current Study

Causality is best established using a randomized controlled experiment, but this approach is clearly impractical in the current context. Where time series data exist, intervention analysis (Enders 2008) provides a quasi-experimental alternative. It uses a multivariate framework to estimate whether, after controlling for other factors, the introduction of an intervention/event is associated with changes in outcomes of interest. Where an intervention is introduced only once, this is an AB design, where the period A is used to establish a baseline, and the period B to estimate the effect of intervention. However, where an effect is detected, the possibility exists that factors omitted from the analysis could explain it. Ordinary Least Squares models are not suited to this task, but a formal time series analysis (e.g. ARIMA) can help to address this (e.g. Nivette et al. 2021). However, if the effect of the intervention can be expected to disappear when the intervention is removed (as would be expected in the case of the routine activity explanation), then alternative designs, such as an ABA, or ABAB design provide a still more robust framework for estimation, since a very particular statistical signature would be expected. We exploit this approach here as there have been three UK lockdowns since the start of 2020, each of which clearly (see below) impacted on people’s (online) activities, generating theoretical expectations as to how levels of online crime should have been affected at different stages of the pandemic. To explain, the timing and relaxation of the COVID-19 restrictions effectively produced an ABABABA design which would be expected to affect the number of suitable targets, or the amount of time they were exposed to risk per unit of time in a predictable way.

**Fig. 1:**
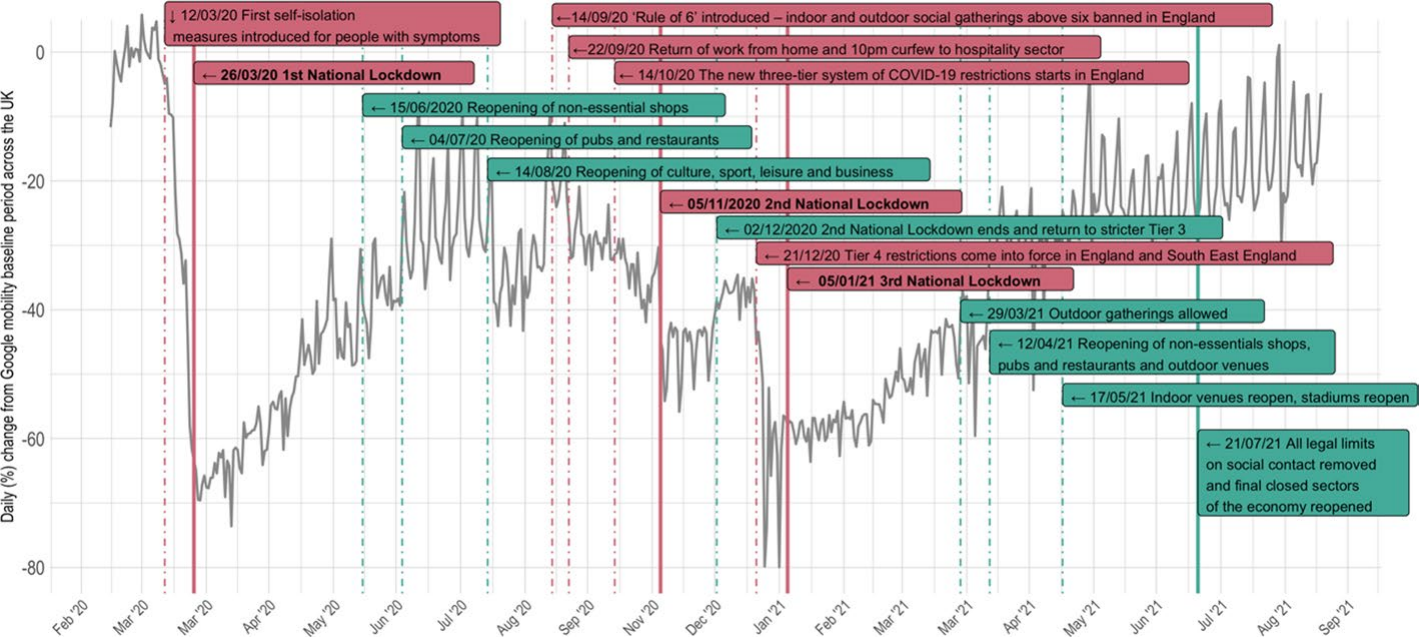
The timing of UK COVID-19 restrictions and Google mobility data for transit stations (the timing of the implementation of policies are shown in red and the relaxation of policies are shown in green. The Google mobility index (shown in grey) was computed by taking the daily average for the UK)

**Fig. 2:**
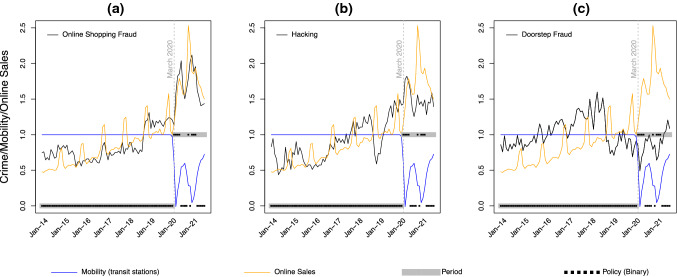
Time series for online shopping fraud (**a**), hacking (**b**) and doorstep fraud (**c**), Google mobility and online sales

That is, the COVID-19 restrictions imposed would be expected to reduce people’s activities in settings outside of the home (see Figs. 1 and 2), but to increase their online activity, including online shopping (see Figs. 1 and 2). In contrast, the lifting or relaxation of restrictions would be expected to lead to a return towards “normal” patterns of activities or an approximation of them. As such, the introduction and lifting of restrictions would be expected to create oscillations in people’s activities on– and off–line. As discussed above, unless there were changes to the security practices that people engaged in online, or there were systematic changes to the guardianship provided online (e.g. from spam/phishing filters, internet service providers, or others), from a routine activity perspective, this would be expected to affect crime opportunities by increasing the number of suitable targets online and hence changing the rate at which motivated offenders could interact (either directly, or via phishing emails or spoofed websites, for example) with them. We are unaware of any research that suggests (and we have no reason to suspect) that there was a change in people’s security practices, or changes to the guardianship provided online during the pandemic. Consequently, we hypothesize that during the pandemic, COVID-19 restrictions (and their relaxation) would have substantially affected people’s routine activities and that monthly variation in levels of online activity should be positively associated with variation in levels of online offenses (H1a). Conversely, we would anticipate that monthly variation in levels of mobility would be negatively associated with levels of online crime (H1b). In the case of doorstep fraud, this has the same motivations as its online equivalents, but we would expect a different trend. The reason for this is that while the precise requirements of government COVID-19 restrictions varied over time, at various stages these imposed strict mobility restrictions that required people to stay at home for all but essential reasons (effectively shopping or exercise), to observe social distancing, to work from home, and to avoid social interactions at other people’s homes or in their gardens. Even support workers, who would previously have visited people’s homes, were required to do so online rather than in person at certain stages of the pandemic period.^1^ Compliance with these restrictions was high—for example, Gimma et al.’s (2022) UK study of people’s mean daily social contacts during the pandemic indicated that these fell from around 11 per day prior to the pandemic to as little as 2 per day during the initial lockdown (these figures were lower for those over the age of 60). With these restrictions in place, and with most people complying with them, doorstep interactions were unusual and those engaged in them would be treated cautiously. As such, UK COVID-19 restrictions limited the rate at which offenders could approach and interact with suitable targets on the doorstep without suspicion. Consequently, we expected doorstep fraud to be positively associated with levels of mobility—declining when restrictions limited movement and increasing as restrictions were eased (H2). This type of fraud thus serves as a non-equivalent dependent variable (Shadish, 2002) which allows us to check that patterns are selective and in line with theoretical expectations (Cook et al., 2002).

## Methodology

### Data

In this section, we describe the key features of the data analyzed and the approach to analysis taken. Additional details regarding the data used and other analyses conducted are provided in an online supporting information (SI) document and reference is made to the latter, where appropriate.

#### Crime Data

To estimate the effect of COVID-19 policies on crime, we use monthly counts of hacking, online– and doorstep–fraud offenses committed within the United Kingdom (UK) for the period 1 January 2014 to 31 August 2021. Data were provided by Action Fraud, which is the UK’s national reporting centre for fraud and cybercrime. Crimes reported to them are classified according to UK Home Office counting rules (Home office 2021) and the data they collect provide the most comprehensive and consistent picture of online crime reported to policing agencies in the UK (for further details of the offenses included, see SI Table S1). Most offenses apply not just to desk and laptop computers but to any device that can run software, such as a smart phone or gaming console.

#### COVID-19 Policy Data

As Fig. 1 shows, there have been three national lockdowns in the UK to date, while several other policies, collectively known as the “Tier” approach, were implemented. To elaborate, during the three national lockdowns^2^ people had to stay at home and minimize social contact while non-essential shops and hospitality businesses had to close. Before the second national lockdown the UK government introduced the first system of tiered restrictions which were in force until the commence of the second national lockdown. During this period, legal restrictions varied across the UK according to government assigned tiers that corresponded to local COVID-19 alert levels (medium, high and very high—the three tiers). Tier 1 prohibited people from socializing in groups of more than six, Tier 2 prohibited people from meeting or visiting people apart from their own household indoors, and Tier 3 prohibited people from mixing with other households both indoors and outdoors. Across all three tiers, shops remained open, but hospitality businesses had to close by 10 pm, and working from home was encouraged. Travel and the use of public transport was also limited for Tiers 2 and 3. Subsequently, following the second national lockdown the government implemented the second tiered system which lasted until the third national lockdown began. Under the second tier system the existing tiers were slightly modified^3^ and ‘Tier four’ level restrictions were introduced across London and other parts of the UK effective from 20 December 2020. The Tier 4 restrictions were essentially lockdown rules as people were asked to stay at home and non-essential shops and hospitality businesses were closed. Collectively, these changes were introduced to limit the spread of the new alpha variant that was circulating during the 2020 Christmas period.

The intensity of the effect of each policy^4^ on people’s activities differed and their effects were not sustained in the way that a simple binary lockdown policy variable, such as that used in previous work, would imply. Consequently, to test the routine activity perspective in a more direct way, we model changes in the “intensity” of COVID-19 restrictions on people’s activities, using two variables. First, we used Google mobility data (see below) to provide a measure of “movement restriction” intensity for the pandemic period that theoretically ranges from zero (no policy) to one. Second, we model changes to people’s online shopping activity (see below).

#### Mobility Data

To estimate changes in mobility, and test H1b and H2, we use Google mobility data concerning the use of transit stations (e.g. underground stations, bus and train stations). For each day, the data show the percentage change in mobility relative to a reference period of 3 January to 6 February 2020. The latter was selected by Google and hence cannot be changed, but we note that changing the reference period would not change the patterns observed, just the absolute percentage change per day. To generate a time series with the same temporal unit of analysis as the crime data, we compute the average index of mobility for each month (See Fig. 2), which ranges from − 1.9 to − 62.7% for the pandemic period. This provides a measure of intensity of the effect of COVID-19 restrictions on people’s routine activities. For the pre-pandemic period, the mobility “intervention” variable is dummy coded to zero. Other mobility indicators (e.g. residential, workplace, and retail and recreation) exist, but as shown in Table 1, the monthly values are highly correlated with each other in general and with the transit measure in particular (See also SI Fig. S2). Consequently, it is the transit indicator that we use in the analyses that follow.

**Table 1:** Correlation matrix for different Google Mobility indices for the pandemic period (all ps < 0.01)

	(1)	(2)	(3)	(4)	(5)
Transit Stations (1)	–				
Work (2)	0.86	–			
Residential (3)	− 0.94	− 0.94			
Parks (4)	0.53	0.21	− 0.48		
Retail & Recreation (5)	0.93	0.84	− 0.97	0.59	
Groceries & Pharmacies (6)	0.77	0.77	− 0.85	0.57	0.86

#### Online Sales Data

To test H1a, online sales data were obtained from the Office for National Statistics (ONS) who generate the Retail Sales Index. The data are collected each month from approximately 5000 British retailers who account for over 90% of all known turnover across the retail sector. The data record the monthly value (at current prices) of estimated online retailing in millions and are available for five categories of online sales (food stores, textile stores, other stores (e.g. chemists, toy stores), household goods stores, and non-store retailers, most of which do not have physical stores) as well as “all” online retail sales. As shown in Table 2, all of the indices are highly correlated in general and with the overall category in particular. Hence, it is the data for the overall category that we use here and the time series for the pandemic period can be seen in Fig. 2 (see also SI Fig. S3).

**Table 2:** Correlation matrix for the different ONS online sales indices (all ps < 0.01)

	(1)	(2)	(3)	(4)	(5)
All Online Sales (1)	–				
Food Stores (2)	0.97	–			
Textiles (3)	0.81	0.80			
Other Stores (4)	0.91	0.93	0.93		
Household Goods (5)	0.96	0.96	0.86	0.95	
Non-Store Retail (6)	0.93	0.90	0.95	0.96	0.94

The data are provided with and without seasonal adjustments, and we use the data without seasonal adjustments, as the former may conceal important short-term changes in the time series. However, as discussed below, the reader should note that the statistical model employed controls for seasonal factors. To use the ONS data as an “intervention” variable which is comparable to that for mobility, we (dummy) code the data for the pre-pandemic period to zero and use the variability in online sales as a measure of the intensity of the effect of COVID-19 policies on online activity for the period February 2020 onwards. While this does not capture all forms of online activity, it provides one indicator of how the pandemic influenced people’s online activities.

## Analytic Strategy

Time series data are not independent, which violates the assumptions of analytic techniques such as OLS regression. Consequently, many existing studies of COVID-19 and urban crime have used Autoregressive Integrated Moving Average (ARIMA) time series methods to model trends. Advantages of this approach include the fact that it enables the estimation of the influence of omitted variables that vary over time, which can reduce errors of statistical inference. Some studies of urban crime during the pandemic have used a univariate approach (e.g. Ashby 2020a, b; Langton et al. 2021; Payne 2020, Payne et al. 2020, 2021; Whelan et al. 2021), examining trends in crime alone, while more recent studies have also used a multivariate interrupted times series approach (e.g. Hodgkinson and Andresen 2020; Mohler et al. 2020; Nivette et al. 2021).

In addition to the independent variables of theoretical interest, ARIMA models have three components with associated parameters that must be calibrated. These components, usually labelled *p*, *d*, and *q*, will now be briefly discussed to make the results that follow easier to interpret. A key assumption that must be met for time series analysis is that the data analyzed are stationary, meaning that the time series has a constant mean and variance over time. If this assumption is violated, the data can be differenced (one or more times) to remove the observed trend. This is captured by the *d* parameter, which is set to one if differencing the data once addresses the problem.

Prior to analysis, we mean centred all continuous variables and tested for stationarity using the Augmented Dickey-Fuller (ADF) test (Enders 2008). For the pre-pandemic period, the time series for the two online offenses (online shopping fraud: ADF Z = − 1.31, *p* = 0.64; hacking: ADF Z = − 1.34, *p* = 0.46) were non-stationary, while that for Door-step fraud was stationary, but only marginally so (ADF Z = − 2.87, *p* = 0.048). First differencing addressed the issue in all cases. The independent variables were also found to be non-stationary for the pandemic period (Google Mobility: ADF Z = − 2.31, *p* = 0.17; Online sales: ADF Z = 1.98, *p* = 0.29). Again, first differencing addressed the issue. In terms of modelling and hypothesis testing, differencing the data has the benefit of removing long-term trends, which means that the estimated coefficients reported in the next section are net of these. In what follows then, all non-stationary variables are first differenced (i.e. d = 1).

Where the variance in a time series is not constant over time (i.e. the data exhibit heteroskedasticity), further transformations may be required. Alternatively, Autoregressive Conditional Heteroskedasticity (ARCH) models can be used to deal with the issue (Enders 2008). For the dependent variables analysed here, Lagrange Multiplier (LM) tests for Heteroskedasticity were statistically significant in all cases (for online shopping fraud: ${\chi }^{2}$(1) = 64.79, *p* < 0.001; for hacking: ${\chi }^{2}$(1) = 49.32 *p* < 0.001; and, door-step crime: ${\chi }^{2}$(1) = 16.71, *p* < 0.001) suggesting the need to test whether ARCH models would be necessary to deal with this issue. Consequently, in what follows LM diagnostic tests were computed for all ARIMA model specifications, and an ARCH model used, where appropriate.

The ARIMA *p* and *q* components relate to the lags used for autoregressive and moving average terms, respectively. The former determines the number of lags of the dependent variable that are included in the ARIMA model, while the latter determines the number of lags of the error term that are included. The inclusion of these terms is intended to remove serial correlation in the time series and consequently errors of statistical inference. Once the number of lags has been determined, coefficients for each lag (and the independent variables of theoretical interest) are estimated using maximum likelihood estimation.

To estimate the ARIMA models, we use the *arimaauto* (Bolotov 2022) function in STATA, which searches for and selects the best ARIMA parameters for a given model. Post-estimation, we test for: (1) serial dependence in the residual errors using the Portmanteau test for white noise; and, (2) the Lagrange Multiplier tests for Heteroskedasticity. Where the latter indicated the presence of heteroskedasticity in the model residual errors we use ARCH time series models.

### Model Specification

We estimate separate models for our two routine activity variables. According to our hypotheses, it is changes to people’s routine activities that should predict changes to online offending (and doorstep crime) during the pandemic. As such, the effects of COVID-19 restrictions (and their relaxation) are predicted to be indirect. That is, while restrictions should impact upon routine activities, it is the latter that should impact upon crime. Of course, changes to COVID-19 restrictions could impact upon crime in other ways. Consequently, to isolate the predictive value associated with our routine activity variables from other potential effects of the restrictions, we include a binary variable that indicates when restrictions were in place and when they were not. This is coded one for periods when lockdowns were implemented, and zero otherwise (prior to differencing). By doing so, our model is intended to estimate the effect of monthly changes to people’s activities on crime net of the overall effect of changes to restrictions.

In addition, we included dummy variables, one for each month, to control for the influence of seasonal effects. As different months have different numbers of days, we also used a continuous variable to account for this. For all analyses, we used the same overall model specification (see Eq. 1) apart from the independent variable of interest and the number of lags used for the AR and MA terms.

1 $$\begin{aligned} \Delta y_{t} & = a_{0} + \mathop \sum \limits_{{i = 1}}^{{11}} \beta _{i} Month_{i} + \beta _{{12}} Days_{t} \\ & \quad + \beta _{{13}} \Delta Intensity_{t} + \beta _{{14}} \Delta Policy_{t} \\ & \quad + \mathop \sum \limits_{{j = 1}}^{p} a_{j} \Delta y_{{t - j}} + \mathop \sum \limits_{{k = 1}}^{q} c_{k} \varepsilon _{{t - k}} + \varepsilon _{t} \\ \end{aligned}$$

where ${\Delta y}_{t}$ is the differenced dependent variable for month *t*; ${a}_{0}$ and the $a, \beta$ and $c$ terms are coefficients to be estimated; ${Month}_{i}$ is a dummy variable for month *i* (the seasonal effect of month is estimated relative to December)^5^; ${Days}_{t}$ captures the numbers of days in month *t*; ${Intensity}_{t}$ is one of the two routine activity variables (e.g. Google mobility) for month *t* (the $\Delta$ term indicates that the data are differenced); ${Policy}_{t}$ is the binary policy variable; the ${y}_{t-j}$ and ${\varepsilon }_{t-k}$ are the autoregressive and moving average terms (for the appropriate number of lags, defined by *p* and *q*), respectively; and, ${\upepsilon }_{t}$ is the residual error at time *t*.

For parsimony, in the text, we report the coefficients for the independent variable of interest, Q-statistics (which indicate whether serial correlation is present in the model residuals), and the log-likelihood value, which provides an indication of the overall model fit. Larger values indicate better model fit. Examples of the coefficients for full models are shown in SI Table S3. Alternative model specifications were tested but produced consistent results. All analyses were conducted in STATA17/MP.

## Results

Table 3 shows descriptive statistics for the key variables analyzed. Consistent with our hypotheses and routine activity theory, relative to the historic period, it is clear that the two online offenses and internet sales were higher during the pandemic, but doorstep crime and mobility were lower. Of course, the comparison of mean values for the two periods is likely to be misleading given that there may be (and are) long term trends in the data.

**Table 3:** Mean monthly values before and during the pandemic

	Pre-COVID-19 Mean	COVID-19 Mean	Total
*Crime variables*			
Online shopping and Auction fraud (s.d.)	3911.5	7859.2	434,864
	(962.1)	(1329.5)	
Hacking (s.d.)	934.4	1592.1	98,463
	(294.7)	(168.7)	
Door to door sales and Bogus tradesmen (s.d.)	468.9	397.3	41,779
	(96.8)	(77.6)	
*COVID-19 policy intensity*			
Google mobility (% change) (s.d.)	–	-37.45 (14.04)	–
Internet sales (£M) (s.d.)	1080	2295	–
	(331)	(411)	
Binary policy variable	0	0.44	–

Raw values shown for Google mobility and Internet sales

Panels a–c in Fig. 2 show mean centre﻿d time series data for the crime, mobility and online sales data. For illustration purposes in Fig. 2, for the pre-COVID-19 period, the Google mobility data take a value of “1” for each month. For the remainder of the time series, the values shown are expressed as a proportion of the maximum percentage change in mobility (a reduction of 62.6%) relative to the baseline. Values closer to one thus represent a return to pre-pandemic levels. For online shopping and doorstep fraud in particular, the observed patterns rather strikingly support routine activity theory. For the former, crime increased during the pandemic and the time series is the mirror image of the Google mobility data and almost identical to that for online sales. Similarly, for door-step fraud, during the pandemic period, the level of crime was lower than the historic period and it tracks levels of mobility almost perfectly. For hacking, there is an initial peak in crime which mirrors the google mobility data and tracks trends in online sales, but levels of hacking do not subsequently exhibit the same peaks observed for online shopping fraud.

As discussed, to test hypotheses, we used ARIMA and Autoregressive Conditional Heteroskedasticity (ARCH) time series models, as appropriate. Table 4 shows the estimated coefficients calculated using the *autoaarima* function for our binary policy variable and our two routine activity variables, along with the optimal ARIMA (p, d, q) specification identified, *Q* statistics, and the log-likelihood statistics. For parsimony, the coefficients for the seasonal fixed effects and other terms are not shown in Table 4 but an example can be found in the online SI Table S3. It is evident that different ARIMA specifications were necessary to provide the best fit to the data in each case, but that after doing so, there was no remaining serial dependence in the residual errors. To explain, the *Q* Statistics were computed using the *wntestq* in STATA which calculates the Portmentau test for white noise. The null hypothesis for this test is that there is no serial dependence in the model residual errors. None of the Q statistics were statistically significant, thereby suggesting that the models were correctly specified in all cases (from this perspective). In terms of the coefficients shown, it is important to note that these are for differenced data and hence should be interpreted as indicating how much the differenced dependent variable changes for a one unit change in the differenced independent variable of interest.

**Table 4:** Time-series analysis (coefficients for seasonal effects and other terms not shown)

	ΔOnline Shopping Fraud	ΔHacking	ΔDoor-Step Fraud
*Mobility Model*			
ΔMobility (s.e.)	− 0.40** (0.08)	− 0.54** (0.09)	0.22* (0.11)
ΔPolicy (s.e.)	− 0.18* (0.06)	− 0.08 (0.08)	− 0.04 (0.08)
ARIMA (p, d, q)	(0, 1, 3)	(5, 1, 1)	(0, 1, 5)
Q-Stat	38.69	24.23	21.25
Log-Likelihood	90.80	57.09	80.13
*Online Sales Model*			
ΔOnline Sales (s.e.)	0.35** (0.08)	0.14 (0.19)	− 0.08 (0.18)
ΔPolicy (s.e.)	− 0.09 (0.06)	0.20* (0.09)	0.14 (0.10)
ARIMA (p, d, q)	(3, 1, 2)	(0, 1, 1)	(0, 1, 5)
Q-Stat	33.47	37.68	24.69
Log-Likelihood	89.61	37.99	76.45

*p < 0.05

**p  <  0.01

In terms of the results, Table 4 shows that after controlling for other factors, including the long-term trend and the timing of policy changes, the estimated coefficients for the mobility variables were statistically significant and in the directions predicted by routine activity theory for all offense types. With respect to doorstep fraud, the Lagrange Multiplier test indicated evidence of heteroskedasticity at lag 7. Consequently, we recomputed the coefficients using an ARCH model specification. This produced the same conclusions (ΔMobility = 0.16, s.e. = 0.05, ΔPolicy = -0.08, s.e. = 0.07, Q-Stat = 29.58, Log-Likelihood = 89.89) providing confidence in the result shown in Table 4. For the online shopping fraud model, the binary policy variable was also statistically significant. As discussed above, this variable was included to help isolate the effects of the routine activity variables and consequently, this result will be discussed no further.

For the online sales activity variable, for online shopping fraud, the estimated coefficient is statistically significant and in the expected direction. However, for hacking and door-step fraud the coefficients are in the expected directions but non-significant. This is consistent with the patterns shown in Fig. 2. For example, for online shopping, the largest increase and largest decline in activity occurred during the middle of the pandemic period. This is also when online shopping fraud peaked and subsequently declined the most, but it is not when hacking peaked. Moreover, the mobility and door-step fraud time series resemble a “w-shaped” signature during the pandemic period,^6^ but for online shopping activity this pattern (or rather than an inverted w-shape) is not observed. For hacking, the binary policy variable was statistically significant suggesting that hacking increased as restrictions were imposed, but as this variable was included as a control variable we discuss this no further.

## Discussion

Different theories of crime emphasize the role of different causal factors. Routine activity theory emphasizes the role of crime opportunity and in particular how people’s routine activities influence these. Furthermore, and perhaps just as importantly, according to the theory, levels of crime may change absent changes to overall levels of offender motivation. Government (restriction) policies introduced to help address the COVID-19 pandemic led to dramatic changes in people’s day-to-day activities which, according to the routine activity perspective, should reduce opportunities for crime in urban environments and increase them online. In this study, we examined how levels of offending for two of the most high-volume forms of online crime, and an urban equivalent (doorstep fraud) changed in the UK during the pandemic and provide a much more direct test of routine activity theory than previous work. Our findings suggest that overall, online shopping fraud and hacking increased, while doorstep fraud did not. Importantly, for each type of crime, changes were not persistent throughout the period study, but there was clear monthly variation. In fact, consistent with H1a and H1b, it appears that levels of online fraud were coupled to patterns of mobility and online activity, increasing as restrictions were imposed, and declining as they were relaxed. This pattern is consistent with what would be expected for the type of intervention analysis reported here if changes in activity patterns were (causally) responsible for changes to online offending, providing clear support for the routine activity perspective. As predicted, monthly variation in doorstep crime was positively associated with patterns of mobility, providing support for H2 and routine activity theory more generally. This finding is important and further increases confidence in the overall findings as the trajectory for doorstep crime was the reverse of that for online crime demonstrating a selective pattern of results that are in line with our hypotheses.

Other researchers have considered the explanatory potential of other criminological theories in the context of the pandemic. For example, Strain theories of crime (Agnew 1992) have been invoked as an alternative theoretical framing for understanding how crime might change during disruptions such as a pandemic (see Nivette et al. 2021). Such theories seek to explain criminal behavior as a consequence of individuals being unable to achieve aspirations and goals (economic or otherwise) through legitimate means, which leads to stress, anxiety and sometimes engagement in crime (Agnew 1992). However, studies intended to test such hypotheses have so far failed to find support for this perspective (Payne and Morgan 2020; Payne et al. 2020b, 2021; Campedelli et al. 2020; Niviette et al. 2021) conclude that the primary mechanism through which the pandemic affected urban crime was through the impact it had on people’s routine activities.

Some (Buil-Gil et al. 2020; Kemp et al. 2021; Nivette et al. 2021) have also suggested that offenders may have adapted during the pandemic, switching from committing crime in urban environments to doing so online. If true, crime committed online would be expected to have increased during the pandemic, but there would be little reason to expect it to decline as restrictions were lifted, which is contrary to our findings. Moreover, as Farrell and Birks (2018) note, the skills required to commit offenses in urban environments are different to those necessary to commit crime online, questioning the logic underpinning such a displacement hypothesis. In short, while alternative perspectives have been proposed to explain changes to patterns of offending during the pandemic, to date the available evidence provides the strongest support for the routine activity explanation.

Returning to the current findings, we noted that for online shopping fraud and door-step fraud, there were multiple peaks and troughs in activity patterns that were replicated (or mirrored) in shape and magnitude almost perfectly in the crime data. For hacking, the initial peak and trough observed in the activity data were observed, but subsequent oscillations were less apparent. Why this form of offending did not exhibit the same second peak that online shopping fraud did is unclear. One possibility is that crime prevention responses to the increase in online crime during the pandemic suppressed this type of crime. This would include, but is not limited to, the UK National Centre for Cyber Security’s (NCSC) COVID-19 response (NCSC 2020). This included take-downs of phishing web-sites, which are used to steal people’s online credentials (e.g. usernames and passwords), and a protective DNS service that prevents people accessing malicious (phishing) domains and IP addresses (including fake Government websites). In 2020, the NCSC took down fifteen times more websites, and blocked more than ten times as many IP addresses than they did in 2019. It was beyond the scope of the current study to explore this further but future work might seek to do so.

More generally, this type of intervention represents an online example of what Clarke and Eck (2003) refer to as “place management” (see also, Eck and Madensen 2018). Place management forms one element of the crime triangle, which is a revision of the original routine activity triangle. It emphasizes not just the roles of the offender, target and capable guardian, but also of those who might act as "handlers” of offenders, and those who do (or should) manage “places” where crimes can occur. Place managers are those who have the legal authority to exert control over a place and in the context of online environments, might include the owner of a website, a service provider that hosts a website, or an organization such as the NCSC which is the UK’s technical authority for cyber threats. Further research might seek to develop our understanding of any changes to place management (e.g. the activity of the NCSC) that occurred during the pandemic and how this may have influenced levels of online crime. It might also consider what other place managers could (and should) do to reduce offending online in general and during times of disruption in particular.

This study is, of course, not without limitations. Chief among these is the fact that much crime committed online goes unreported (ONS 2020). As such, it is possible that some of the patterns reported here might be explained by changes in victim reporting behavior. While this cannot be ruled out entirely, we do not think this is the case because we observe quite distinct statistical signatures in the data that would be expected on the basis of routine activity theory, but not if changes to reporting behavior had simply increased over the course of the pandemic.

While we have not drawn attention to the absolute volume of offenses analyzed, these are worth consideration. Estimates from the Crime Survey of England and Wales suggest that about half of all crime is now online, and as noted much crime committed online goes unreported (ONS 2020). What this suggests is that the volume of offending discussed in this paper likely represents only the tip of the iceberg.

Put bluntly, cybercrime accounts for a substantial amount of all crime and this share appears to have increased during the pandemic. If the increases in online activity observed during the pandemic persist, absent changes in guardianship online, so too might the increase in online fraud. This has to be considered against a backdrop in which many types of urban crime have declined in recent decades and during the pandemic period (Nivette et al. 2021). However, just like urban crime, the term “cybercrime” includes an array of offenses, each with their own opportunity structure. Accordingly, what is needed is the detailed analysis of how the opportunity structure changed for different forms of cybercrime. Furthermore, for traditional urban crime there is a growing literature on “What Works” to reduce it, and a growing body of systematic reviews that are required to synthesize it (Weisburd et al. 2016). This contrasts with the literature on cybercrime. For example, a recent review of the literature (Brewer et al. 2019) concluded that “to date there has been little to no research evaluating the effects of crime prevention initiatives on cybercrime” (p. 125). The contrast is startling and suggests the need for the academic and policy communities to engage more with the changing nature of crime and “what works” to reduce it.

## Supplementary Information

Below is the link to the electronic supplementary material.

Supplementary file1 (DOCX 338 KB)

## Footnotes

1 See, https://www.redcross.org.uk/-/media/documents/about-us/research-publications/health-and-social-care/life-after-lockdown-tackling-loneliness-among-those-left-behind-report.pdf.

2 The first lockdown commenced on 26 March 2020 but restrictions were gradually lifted from 11 May 2020. The second lockdown began on 5 November 2020 and lasted until 2 December 2020, while the third lockdown started on 5 January 2021 with restrictions being gradually lifted from 8 March 2021 onwards.

3 Under the new tier system, large gatherings (which were previously prohibited across all three tiers) were allowed with restrictions for Tiers 1 and 2. For Tier 3, hospitability businesses had to be closed and operate only by delivery (if available). For Tiers 1 and 2, The opening hours of hospitability businesses were extended to 11 pm instead of 10 pm as per the previous system. A detailed description of the Tier system is available at https://researchbriefings.files.parliament.uk/documents/CBP-9068/CBP-9068.pdf.

4 UK COVID-19 policy trackers are available from https://www.health.org.uk/news-and-comment/charts-and-infographics/covid-19-policy-tracker. and https://www.instituteforgovernment.org.uk/charts/uk-government-coronavirus-lockdowns.

5 The monthly dummy variables and the variable that captured the number of days per month were, of course, all stationary and were not differenced. However, we note that differencing these variables produced the same pattern of results as those reported in the paper.

6 For door-step fraud the signature is a little more jittery.
