# Supplementary material for: The Effect of COVID-19 Restrictions on Routine Activities and Online Crime
Source: J Quant Criminol. 2022 Dec 8:1–20. Online ahead of print. doi: 10.1007/s10940-022-09564-7 (PMC9735226; doi:10.1007/s10940-022-09564-7)
Supplement: Supplementary file 1 — Supplementary file1 (DOCX 338 KB) [file 10940_2022_9564_MOESM1_ESM.docx]

**Supporting Information** for **“**The effect of COVID-19 restrictions on routine activities and online crime” (Shane D Johnson and Manja Nikolovska, Dawes Centre for Future Crime, University College London)

[**Introduction** 2](#_Toc118728811)

[**1. Crime Data** 2](#_Toc118728812)

[Table S1 Types of fraud examined as per the UK Home office counting rules (Jan 2014- Aug 2021) 2](#_Toc118728813)

[**2. Characteristics of the time series** 3](#_Toc118728814)

[**Fig S1** Mean centred and differenced crime time series data for online sales fraud 3](#_Toc118728815)

[**3. Google mobility indices** 3](#_Toc118728816)

[**Fig S2** Time series for the different Google Mobility indices 4](#_Toc118728817)

[**4. Full model results** 4](#_Toc118728818)

[**Table S3** Estimated coefficients for online shopping fraud using ARIMA models and differenced data 5](#_Toc118728819)

[**5. Online Sales Data** 5](#_Toc118728820)

[**Fig S3** Online sales by type of retail 6](#_Toc118728821)

# **Introduction**

This Supporting Information (SI) document provides further details of the data analyzed along with examples of analyses that could not be reported in full in the main paper.

# **1. Crime Data**

Data were provided by Action Fraud which is the UK’s national reporting centre for fraud and cybercrime. Crimes reported to Action Fraud are classified according to UK Home Office counting rules and the data they collect provide the most comprehensive and consistent picture of online crime reported to policing agencies in the UK. Table S1 provides details of the offence types and their frequency for the period studied.

| Table S1 Types of fraud examined as per the UK Home office counting rules (Jan 2014- Aug 2021) | | | |
| --- | --- | --- | --- |
| Type | **Code** | **Definition** | **Count** |
| Online shopping and auctions | NFIB3A | Shopping and Auction fraud involves fraud attributable to the misrepresentation of a product advertised for sale through an Internet auction site or the non-delivery of products purchased through an Internet auction site; or buyers using fraudulent methods to purchase goods or services from legitimate websites for personal gain. | 434,864 |
| Hacking | (NFIB52B & NFIB52C) | Computer Hacking is the unauthorised modification of the contents of any computer or any device using operating software accessible online, for example games consoles and smart phones. It is usually committed by persons unlawfully accessing the computer/device, but it can be committed by persons with lawful access to the computer/device as well. It is the deliberate targeting of a specific computer/device by the offender. | 98,463 |
| (Personal) | NFIB52B | A personal computer is any individual computer or operating software device accessible online that is not a server. | 29,407 |
| (Social media and Email) | NFIB52C | All forms of individual e-mail accounts and all forms of individual social media, for example twitter and Facebook. It includes personal as well as companies or organizations individual accounts. This fraud should not be viewed as limited to desk or lap top computers, it can include any device using operating software device accessible online. | 69,056 |
| Door to Door Sales and Bogus Tradesmen | NFIB3C | Fraud by False Representation, involving an offender who dishonestly makes a false representation (by visiting a victim’s home) and intends to defraud the victim, by making the representation to make a gain for himself or another, or to cause loss to another or to expose another to risk of loss. | 41,779 |

# **2. Characteristics of the time series**

Prior to analysis, we tested for stationarity in all of the time series variables using the Augmented Dickey-Fuller (ADF) test (Enders, 2004) and data for the longest time period (i.e. the period before or during the pandemic) for which data were available. For the crime data, the pre-pandemic period provided the longest time series. In this case, the time series for the two online offences (online shopping fraud: ADF z=-1.31, p=0.64; hacking: ADF z=-1.34, p=0.46) were non-stationary, while that for Door-step fraud was stationary, but only marginally so (ADF z=-2.87, p=0.048). First differencing (see Fig S1) addressed the issue in all cases (ADF z=-10.69, p<0.0001; ADF z=-11.93, p<0.0001; and ADF z=-10.42, p<0.0001 for online shopping fraud, hacking and doorstep fraud, respectively).

**Fig S1** Mean centred and differenced crime time series data for online sales fraud (panel a), hacking (panel b) and door-step fraud (panel c)

The independent variables were also found to be non-stationary for the pandemic period (Google Mobility: ADF Z=-2.31, p=0.17; Online sales: ADF Z=-1.98, p=0.29). Again, first differencing addressed the issue (Google Mobility: ADF Z=-3.06, p<0.03; Online sales: ADF Z=-3.21, p<0.02).

# **3. Google mobility indices**

Google mobility data is generated using anonymized information from mobile phone users that have enabled the location history on a device. The data represent the daily percentage change in the use of different types of places relative to a baseline period (3 January to 6 February 2020). The baseline period was determined by Google. In this paper, we take the mean for each month as the crime data are only available at this level of aggregation. In the main paper, we use Google mobility data for transit stations. Fig S2 shows the time series for the six different indices of mobility generated by Google. They show that during the pandemic, patterns of activity around the home, at parks and sites of recreation increased, while activity at other locations declined. It is also clear that most indices are highly correlated with each other.

## **Fig S2** Time series for the different Google Mobility indices


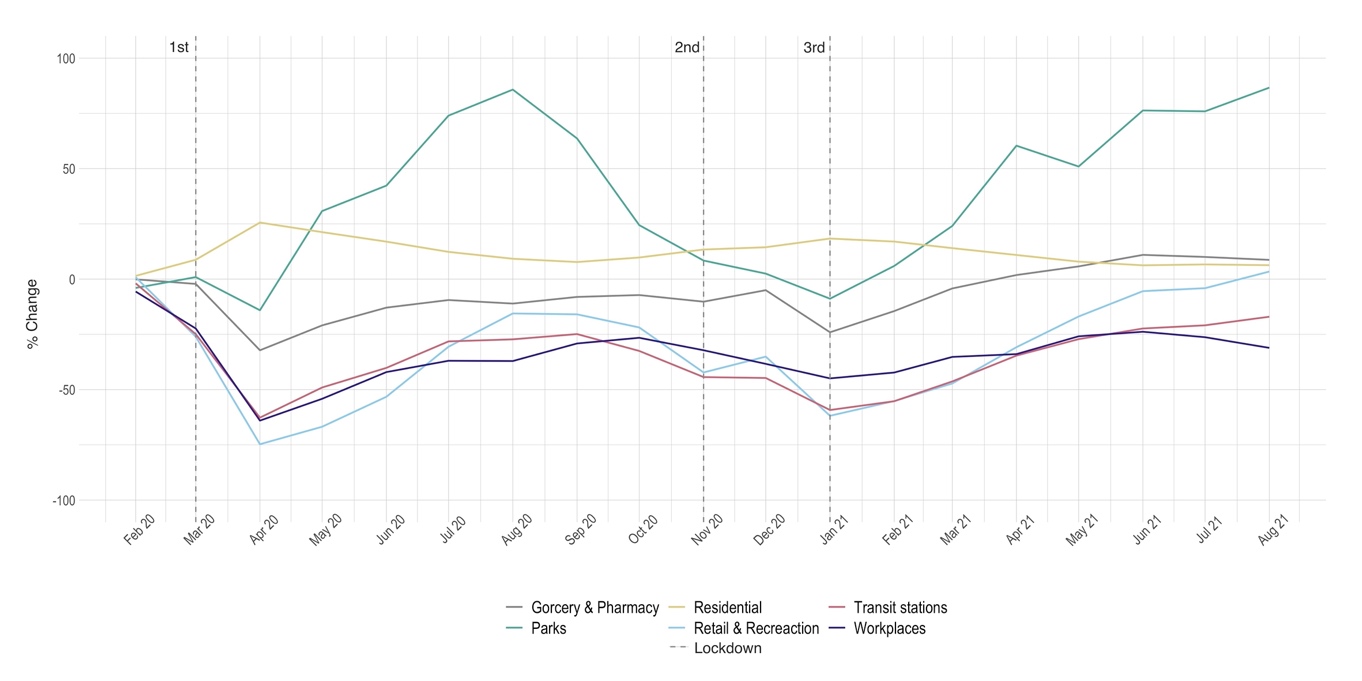


# **4. Full model results**

As noted in the main text, we estimate ARIMA models using the *arimaauto* (Bolotov, 2022) function in STATA, which searches for and selects the best ARIMA parameters for a given model. Post-estimation, we test for: 1) serial dependence in the residual errors using the Portmanteau test for white noise; and, 2) the Lagrange Multiplier tests for Heteroskedasticity. Where the latter indicated the presence of heteroskedasticity in the model residual errors we use ARCH time series models.

In the main text, we report the estimated coefficients for only the key independent variables of interest. This is because the other variables are included as control variables and are not of theoretical interest in and of themselves. For the purposes of illustration, Table S3 shows the estimated coefficients for all variables for the models for which online sales was the key independent variable of interest. As in the main text, the dependent and key independent variables were differenced prior to analysis to make them stationary and we report the estimated coefficients for the best fitting ARIMA specification. The monthly dummy variables were used to estimate seasonal effects and are estimated relative to the month of December.

The overall trends were that, relative to December, (differenced) monthly levels of online shopping fraud tended to be lower, while (differenced) monthly levels of hacking and door-step fraud tended to be higher. However, with a few exceptions, after controlling for other factors, the differences were not statistically significant. The number of days in the month was also not statistically significant for any of the models shown. In Table S3, the monthly dummy and days of the months variables were not differenced because these variables were, of course, stationary. For completeness, we reran the models after first differencing these variables. All substantive findings were consistent with those reported in Table S3 and in the main text. For example, for the online shopping fraud model, the estimated coefficient for the ΔInternet Sales variable was 0.37 (s.e.=0.08, p<0.0001), and the estimated coefficient for the ΔPolicy variable was -0.09 (s.e.=0.06, p>0.14).

## **Table S3** Estimated coefficients for online shopping fraud using ARIMA models and differenced data

|  | Δ Online Shopping Fraud | |  | Δ Hacking | |  | ΔDoor-step Fraud | |
| --- | --- | --- | --- | --- | --- | --- | --- | --- |
|  | b | s.e. |  | b | s.e. |  | b | s.e. |
| Δ Internet Sales | 0.35* | 0.08 |  | 0.14 | 0.19 |  | -0.08 | 0.18 |
| Δ Policy | -0.09 | 0.06 |  | 0.20* | 0.09 |  | -0.14 | 0.10 |
| Month Dummy:  Jan | -0.34 | 0.16 |  | 0.24 | 0.14 |  | 0.11 | 0.09 |
| Feb | -0.25 | 0.26 |  | 0.04 | 0.91 |  | 0.30 | 0.42 |
| Mar | -0.04 | 0.14 |  | 0.13 | 0.13 |  | 0.19* | 0.08 |
| Apr | -0.15 | 0.16 |  | 0.21 | 0.32 |  | 0.11 | 0.13 |
| May | -0.03 | 0.13 |  | 0.07 | 0.09 |  | 0.20* | 0.10 |
| June | -0.09 | 0.16 |  | 0.08 | 0.33 |  | 0.19 | 0.17 |
| July | -0.06 | 0.12 |  | 0.24* | 0.11 |  | 0.16 | 0.10 |
| Aug | -0.14 | 0.11 |  | 0.03 | 0.12 |  | 0.06 | 0.07 |
| Sept | -0.12 | 0.16 |  | 0.11 | 0.33 |  | 0.17 | 0.16 |
| Oct | 0.06 | 0.13 |  | 0.25* | 0.09 |  | 0.07 | 0.08 |
| Nov | -0.10 | 0.16 |  | -0.02 | 0.30 |  | 0.16 | 0.19 |
| Dec (Reference) | - | - |  | - | - |  | - | - |
| Days in Month | -0.05 | 0.09 |  | 0.14 | 0.19 |  | 0.08 | 0.14 |
| Constant | 1.46 | 2.71 |  |  |  |  |  |  |
| AR(1)  AR(2)  AR(3) | 1.08*  -0.42  -0.37* | 0.20  0.24  0.15 |  | -  -  - | -  -  - |  | -  -  - | -  -  - |
| MA(1)  MA(2)  MA(3)  MA(4)  MA(5) | -1.33*  0.76*  -  -  - | 0.26  0.22  -  -  - |  | -0.47*  -  -  -  - | 0.10  -  -  -  - |  | -0.34*  -0.19  0.18  0.21  -0.42* | 0.13  0.12  0.13  0.17  0.17 |
|  |  |  |  |  |  |  |  |  |
|  |  |  |  |  |  |  |  |  |
| Log-Likelihood | 89.61 |  |  | 37.99 |  |  | 76.45 |  |

*p<0.05 **p<0.01

# **5. Online Sales Data**

Data concerning online sales for Great Britain are published by the Office for National Statistics as part of the Retail Sales Index^[[1]](#footnote-2)^. The data are collected each month from approximately 5,000 British retailers who account for over 90% of all known turnover across the retail sector. The data record the monthly value (at current prices) of estimated online retailing in millions. In the main text, we use the data for all online retail sales but for completeness, in Fig S3 we show the data for the five categories of sales for which data are collected. These are food stores, household goods stores, textile, clothing and footware stores, non-store retailers (the majority of which are online retailers that do not have physical stores^[[2]](#footnote-3)^), and “other non-food” retailers which include chemists, toy stores and sports equipment stores.

## **Fig S3** Online sales by type of retail


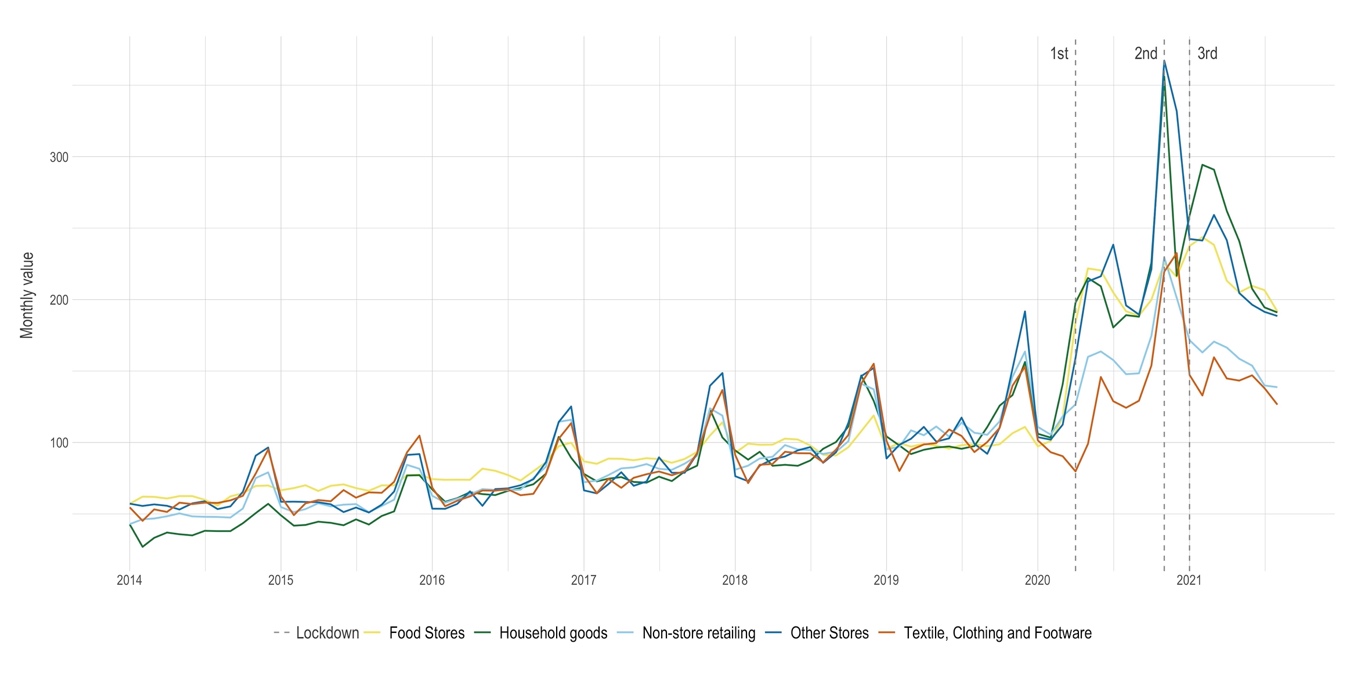


1. See, https://www.ons.gov.uk/businessindustryandtrade/retailindustry/methodologies/retailsalesindexrsiqmi [↑](#footnote-ref-2)
2. https://www.ons.gov.uk/businessindustryandtrade/retailindustry/bulletins/retailsales/september2021 [↑](#footnote-ref-3)
